# Mutant Prourokinase with Adjunctive C1-Inhibitor Is an Effective and Safer Alternative to tPA in Rat Stroke

**DOI:** 10.1371/journal.pone.0021999

**Published:** 2011-07-14

**Authors:** Simone Tomasi, Paolo Sarmientos, Giada Giorda, Victor Gurewich, Alessandro Vercelli

**Affiliations:** 1 Neuroscience Institute Cavalieri Ottolenghi (NICO), San Luigi Gonzaga Hospital, Orbassano, Turin, Italy; 2 Department of Anatomy, Pharmacology and Forensic Medicine, University of Turin, Turin, Italy; 3 Primm s.r.l., San Raffaele Biomedical Science Park, Milan, Italy; 4 Vascular Research Laboratory, Beth Israel Deaconess Medical Center, Boston, Massachusetts, United States of America; Julius-Maximilians-Universität Würzburg, Germany

## Abstract

A single-site mutant (M5) of native urokinase plasminogen activator (prouPA) induces effective thrombolysis in dogs with venous or arterial thrombosis with a reduction in bleeding complications compared to tPA. This effect, related to inhibition of two-chain M5 (tcM5) by plasma C1-inhibitor (C1I), thereby preventing non-specific plasmin generation, was augmented by the addition of exogenous C1I to plasma *in vitro*. In the present study, tPA, M5 or placebo +/− C1I were administered in two rat stroke models. In Part-I, permanent MCA occlusion was used to evaluate intracranial hemorrhage (ICH) by the thrombolytic regimens. In Part II, thromboembolic occlusion was used with thrombolysis administered 2 h later. Infarct and edema volumes, and ICH were determined at 24 h, and neuroscore pre (2 h) and post (24 h) treatment. In Part I, fatal ICH occurred in 57% of tPA and 75% of M5 rats. Adjunctive C1I reduced this to 25% and 17% respectively. Similarly, semiquantitation of ICH by neuropathological examination showed significantly less ICH in rats given adjunctive C1I compared with tPA or M5 alone. In Part-II, tPA, M5, and M5+C1I induced comparable ischemic volume reductions (>55%) compared with the saline or C1I controls, indicating the three treatments had a similar fibrinolytic effect. ICH was seen in 40% of tPA and 50% of M5 rats, with 1 death in the latter. Only 17% of the M5+C1I rats showed ICH, and the bleeding score in this group was significantly less than that in either the tPA or M5 group. The M5+C1I group had the best Benefit Index, calculated by dividing percent brain salvaged by the ICH visual score in each group. In conclusion, adjunctive C1I inhibited bleeding by M5, induced significant neuroscore improvement and had the best Benefit Index. The C1I did not compromise fibrinolysis by M5 in contrast with tPA, consistent with previous *in vitro* findings.

## Introduction

Stroke is the second most common cause of death worldwide [1] of which more than 80% are thromboembolic in origin. Tissue plasminogen activator (tPA) is the only pharmacological reperfusion treatment approved for ischemic stroke [2]. However, its utilization has been hampered by significant problems which include a narrow treatment window (3–4.5 hours), limited efficacy, since only a sub-optimal dose (0.9 mg/Kg) can be administered due to the risk of iatrogenic intracranial haemorrhage (ICH) [3]–[11]. As a result, only an estimated 2-5% of ischemic stroke patients are currently treated with tPA in the US [12], though the number is higher in dedicated Stroke Units.

Single-chain urokinase plasminogen activator (prouPA), the other natural plasminogen activator, was tested in stroke by a single study [13]. ProuPA is a zymogen with little fibrin affinity but has an equivalent fibrin specificity to tPA [14]. An intra-arterial (ia) route of administration was used, but with a six hours treatment window. The ia route was chosen because previous studies showed that at therapeutic concentrations in plasma, prouPA was vulnerable to non-specific activation to two-chain urokinase (tcuPA). As a result, its fibrin-specificity was lost resulting in a haemorrhagic diathesis due to degradation of clotting factors like fibrinogen [15]. This activation of prouPA to tcuPA in plasma, instead of only on the fibrin clot, undermined its therapeutic exploitation and resulted in prouPA not being approved by the European Agency for the Evaluation of Medicinal Products EMEA. At the same time, prouPA had certain advantageous properties, which included a much lower rate of coronary reocclusion than tPA, no associated procoagulant haematological effects, and a low mortality [16], [17].

Therefore, structure-function and mutagenesis studies were undertaken in an attempt to overcome this problem with native prouPA. These led to the development of a single site-directed (Lys300→His) prouPA mutant, M5, which was significantly more stable in plasma at therapeutic doses [18], [19], but otherwise retained the basic mechanism of action of prouPA. Two thrombolytic studies with M5 in dogs with venous or arterial thrombi showed that it was as effective as tPA but caused ten-fold less bleeding from injury sites [20], [21]. In the second of these studies, an unusual plasma inhibitor of two-chain M5 (tcM5) was identified which helped explain the low incidence of bleeding complications. This inhibitor, by inactivating tcM5, prevented non-specific plasmin generation responsible for a bleeding diathesis. The plasma inhibitor responsible was identified to be complement C1-inhibitor (C1I) [20].

In human plasma clot lysis studies with M5, the same inhibition of tcM5 as in the dogs was observed [20], [22], and the addition of exogenous human C1I or recombinant C1I (rhC1I) further increased M5 stability without compromising clot lysis. As a result, higher M5 doses achieving optimal rates of lysis were possible without inducing fibrinogenolysis [22], [23]. These *in vitro* findings suggested that adjunctive C1I, which was recently approved for treatment of hereditary angioedema, might be used to promote both the efficacy and safety of thrombolysis with M5.

In the present study, this hypothesis was tested for the first time *in vivo*, using rat stroke models of irreversible and thromboembolic cerebral ischemia. Preliminary *ex vivo* studies in our laboratory revealed that the endogenous C1I in rat plasma, in contrast to other species, failed to complex with tcM5 and did not inhibit tcM5 activity. This made the rat a virtual C1I knockout animal for M5, thereby making it especially vulnerable to haemorrhagic side effects of M5 and, by the same token, very sensitive to the effects of human C1I. A comparison with tPA and placebo, in presence or absence of human C1I, was then carried out.

## Results

### Laboratory analysis

**Figure 1:**
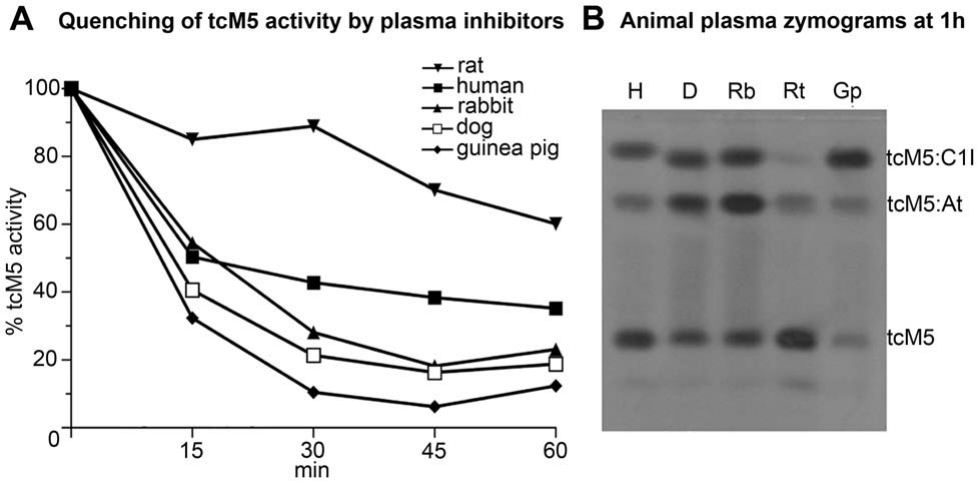
Quenching of tcM5 activity by inhibitors in plasmas from different species. A. tcM5 (10 µg/mL) incubated (37°C, 1 h) in plasma and activity monitored with uPA enzymatic substrate (S2244). Rat plasma induced little tcM5 inhibition, in contrast to the other plasmas. B. After 1 h of incubation, each plasma sample was examined by zymography. No tcM5 complex with C1I, and only a faint complex with AT formed in rat plasma, in contrast to the other species.

Incubation of tcM5 showed quenching of activity within 15 min in the plasma of each species except the rat (Figure 1). This finding was mirrored by the absence of tcM5∶C1I complexes visible on zymography at the end of incubation. Only a faint complex with anti-thrombin was seen, indicating that rat plasma had little endogenous tcM5 neutralizing activity of any kind necessary to inhibit the non-specific effects of M5. This made the rat a functional C1I knockout species with respect to M5.

### Part I (permanent ischemia): neuropathological evaluation

**Figure 2:**
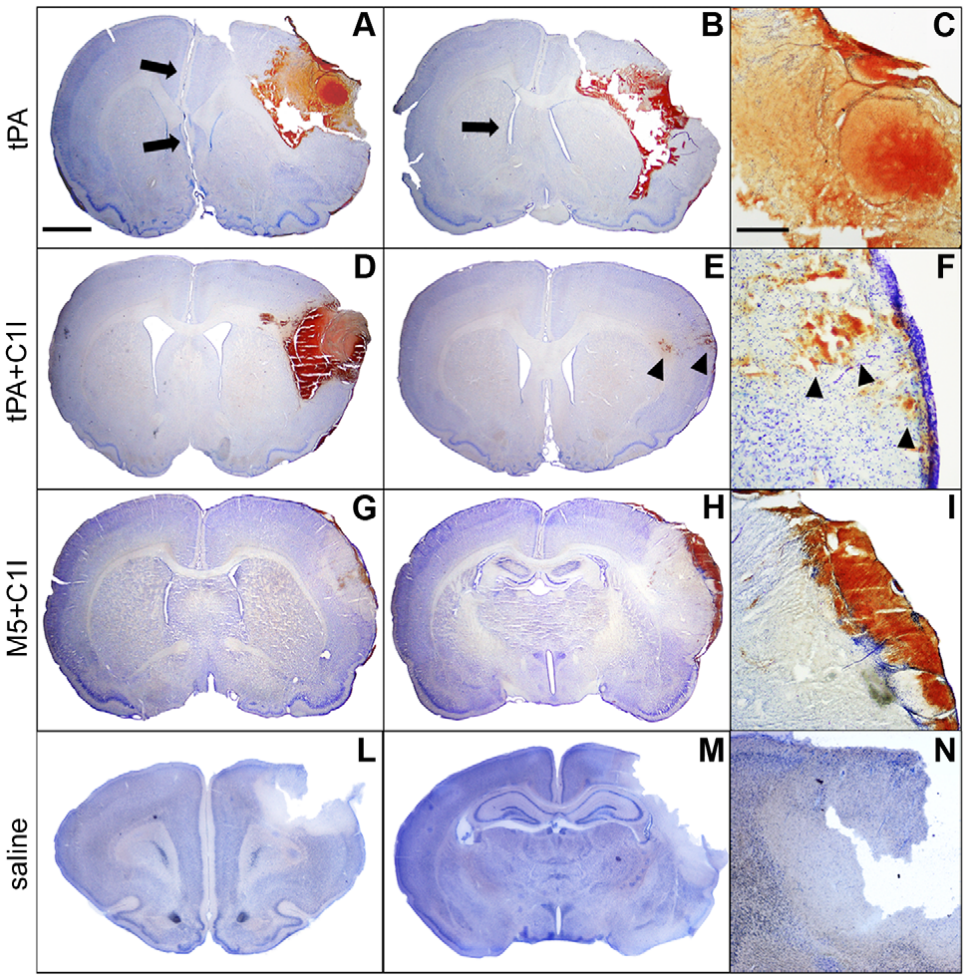
(Part I): representative brain sections from Groups 1, 2,4. A–C: Rat which died from tPA showing extensive hemorrhagic infiltration with edema compression (arrows) of the ischemic hemisphere (enlarged in C). D: Diffuse hemorrhagic transformation of ischemic cortex with edema compression in a Group 2 rat which died of ICH in a few hours. E–F: Sections 24 h after tPA+C1I infusion showing blood within the ischemic core (arrowheads), but no infiltration below the cortical surface (details in F). G–I: Thick surface hematoma above ischemic area, but no major blood infiltration in a M5+C1I rat. This Group showed the least bleeding with no alteration of brain architecture. L–M: ischemic sections from saline group, showing no blood infiltration within the infarct areas (details in N). Scale bars: 2 mm in A–B–D–E–G–H–L–M and 500 µm in C–F–I–N. Group 3 (M5 alone) rats not shown due to extensive disruption of brain by ICH.

Group 1 (tPA): ICH occurred starting at ∼2 h from which 4 of 7 rats died within 12 hours. Extensive hemorrhagic infiltration of the ischemic hemisphere was seen on examination. Edema and ICH compressed the contralateral intact hemisphere as shown (Figure 2A–C). The 3 rats that survived also showed gross ICH on the brain surface and interhemispheric fissure. Group 2 (tPA+C1I): Only 2 of 8 rats died from ICH, and these showed severe disruption of the ischemic cortex and underlying structures with the lateral ventricle invaded by blood (Figure 2D). In the remaining 6, spotty parenchymal hemorrhage was seen in 3 and the cytoarchitecture was preserved in all of them (Figure 2E–F). Parenchymal blood infiltration was diffuse but was confined to the ischemic areas. Group 3 (M5): Only 4 rats were in this group for the reasons described above. As anticipated by the findings shown in the Figure 1, the rat is an unrepresentative species in which excessive bleeding by M5 could be anticipated. In fact, 3 out of 4 rats died of ICH within 12 hours of the infusions and brain sections were not obtained from any animal in this group due to extensive necrosis and blood infiltration, which disrupted the brain architecture (see Figure S1 B). Even in the one surviving rat, extensive blood infiltration of the ischemic core, lateral ventricles and subarachnoid spaces was seen, which caused severe compression of the parenchyma with shift from the midline. The results in this group illustrated the importance of C1I in the prevention of hemorrhagic side effects by M5. Group 4 (M5+C1I): With the addition of C1I, only 1 of 6 rats died of ICH at 12 hours. This was the lowest mortality among the groups, but the difference did not reach statistical significance. Histologically, bleeding was mild and brain architecture was preserved in all the surviving rats (Figure 2G–I). Group 5 (saline) and 6 (C1I): No deaths or ICH occurred (Figure 2L–N, Table 1).

**Table 1:** Comparison of infarct extent, brain edema, neurological score and hemorrhagic complications following thrombolysis by tPA or M5±C1I.

	mean±SEM	Group 1	Group 2	Group 3	Group 4	Group 5	Group 6
		tPA	tPA+C1I	M5	M5+C1I	saline control	saline+C1I
Part I	*N*	*7*	*8*	*4*	*6*	*5*	*5*
	treatment protocol	alteplase 10 mg/kg	C1I 100 IU/kg	M5 15 mg/kg	C1I 100 IU/kg	saline	C1I 100 IU/kg
			alteplase 10 mg/kg		M5 15 mg/kg		
	% animals died due to bleeding (n)	57.1 (4)	25 (2)	75 (3)	16.6 (1)	0 (0)	0 (0)
	% animals with parenchymalhemorrhage (n)	71.4 (5)	50 (4)	75 (3)	33.3 (2)	0 (0)	0 (0)
	% animals with surface hematomas (n)	71 (5)	75 (6)	75 (3)	66.6 (4)	0 (0)	0 (0)
	total hemorrhage (% 10x m.f.)	38.1±9.9	21.1±8.4	-	7.7±2.2	2.5±0.8	
	subarachnoidal hemorrhage (% 10x m.f.)	21.7±5.7	10.6±3.3	-	5.6±2.3	1.3±0.3	-
	intracortical hemorrhage (% 10x m.f.)	10.6±3.7	6.4±3.2	-	1.7±0.2	1.1±0.6	-
	subcortical hemorrhage (% 10x m.f.)	5.8±2.5	4.1±2.9	-	0.3±0.2	0.1±0.06	-
Part II	*N*	*6*	*4*	*6*	*6*	*5*	*4*
	treatment protocol	alteplase 5 mg/kg	rhC1I 180 IU/kg	M5 15 mg/kg	rhC1I 180 IU/kg	saline	saline
			alteplase 5 mg/kg		M5 15 mg/kg		rhC1I 180 IU/kg
	mean ischemic volume (mm^3^)	85±19	142±15	99±41	88±30	213±28	228±13
	mean % ischemia	8.5±1.7	14.4±1.1	9.4±3.6	8.7±3.0	19.9±2.2	21.7±0.9
	mean % ischemic brain salvage following thrombolysis	60.1±8.9	37.5±6.8	53.5±19.3	61.3±13.1	0±13.2	0±5.9
	mean edema volume (mm^3^)	56±17	35±18	51±16	36±11	69±18	74±22
	mean % edema	5.6±2.0	3.4±1.7	4.9±1.4	3.6±1.1	6.5±1.6	6.7±1.6
	neuroscore at 2 h	3.0±0.3	3.5±0.3	3.2±0.2	3.3±0.2	3.2±0.4	3.5±0.3
	neuroscore at 24 h	2.5±0.3	2.5±0.3	2.5±0.6	2.0±0.4	2.8±0.4	3.0±0.0
	ICH visual score	2.5±0.6	1.8±0.5	2.3±0.5	1.7±0.3	1.2±0.2	1.3±0.3
	Benefit Index	0.24±0.05	0.21±0.06	0.23±0.08	0.37±0.10	-	-

#### Part I Summary

ICH and mortality were high with both activators alone, but highest with M5 consistent with the absence of C1I effect in rat plasma. C1I pretreatment reduced ICH and mortality dramatically, confirming its importance in preserving the fibrin specificity of M5 (Group 2). C1I also reduced ICH with tPA, but this was related to inhibition of fibrinolysis, as evidenced by the results in Part II. Semiquantitative measurement of bleeding showed that C1I significantly reduced ICH by M5 compared to tPA (p = 0.02), whereas the C1I reduction of ICH by tPA did not reach significance (p = 0.14) (see *Intracranial hemorrhage* section below).

### Part II (thromboembolic ischemia)

#### Functional outcome

**Figure 3:**
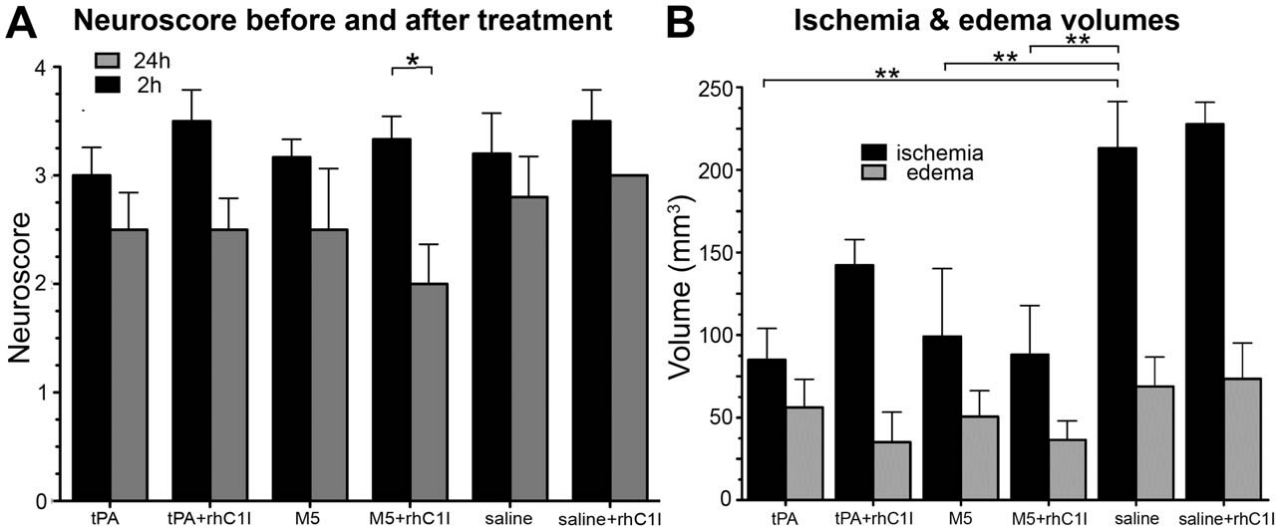
(Part II) Neuroscores (A) and Ischemic and Edema volumes (B). A: Neuroscore pre-(2 h) (black) and post-treatment (24 h) (gray) of thromboembolic stroke. Only Group 4 (M5+C1I) showed significant (*p<0.05) post-treatment functional recovery. B: Ischemia volumes (black) in all groups. Differences were significant (*p<0.05, **p<0.01) from control in all but Group 2 (tPA+C1I). Edema volumes (gray) in all groups. In Group 2 (tPA+C1I), C1I increased the ischemic volume but decreased the edema volume, showing an unusual discordant relationship.

All rats survived for 24 hours after stroke except for 1 out of 6 given M5 alone. This one died of lethal ICH 8 hours after treatment, which was associated with profuse rebleeding from the tail, the only animal in which this was seen. The pretreatment neuroscore at 2 h ranged from 3.0–3.6 in the 6 groups, whereas at 24 h it ranged from 2.0–3.0 (Table 1). Group 4 had the lowest score at 24 h and was the only group where the difference between 2 h and 24 h was significant (p = 0.016) (Figure 3A).

#### Ischemic volumes

**Figure 4:**
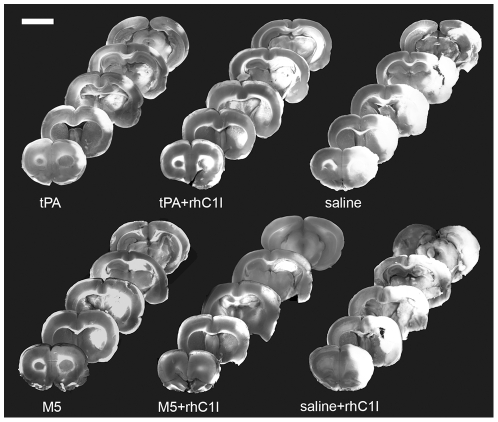
(Part II): Brain infarct zones from representative rats in each group. Five antero-posterior TTC stained 2 mm-thick coronal sections from a representative animal from each group. Infarct areas in cerebral cortex, hippocampus and striatum appear white. The least amounts of infarction were with tPA, M5, and M5+rhC1I. Scale bar: 5 mm.

The ischemic areas were readily identified after TTC staining, facilitating measurement (Figure 3B and Figure 4). Compared to their respective controls (Groups 5 & 6), thrombolysis reduced ischemic volumes significantly and to a similar extent with tPA (Group 1, p = 0.004), M5 (Group 3, p = 0.009) and M5+rhC1I (Group 4, p = 0.004), with brain salvage being 60%, 54% and 61% respectively. This showed that the fibrinolytic effect of tPA alone and M5 with or without rhC1I was comparable. With the addition of rhC1I to tPA (Group 2), the ischemic volume increased ∼40% (85→142 mm^3^), indicating an inhibition of fibrinolysis by the C1I. As a result, brain salvage was no longer significant (p = 0.12) in this group. By contrast, the combination of rhC1I with M5 (Group 4) was associated with a further, small reduction in ischemic volume (99→88 mm^3^) (Figure 3B, Table 1).

#### Edema Volumes

Among the treatment groups, the edema volume with tPA was the highest (56±17) as compared to 36±11 for M5+C1I. With the addition of rhC1I to tPA, the edema volume was reduced to 35±18. This inhibition of tPA-mediated edema by rhC1I was surprising since it was accompanied by a ∼40% increase in ischemic volume, which would have been expected favor edema formation. Since the rhC1I had no effect on edema formation in control groups 6 vs. 5, the rhC1I effect could not be attributed to an inhibition of BBB disruption by ischemia. Instead, the findings implicate tPA-mediated BBB disruption as the C1I target (Figure 3B, Table 1).

### Intracranial Hemorrhage (Part I and II)

#### ICH mortality (Part I)

Out of the 14 rats in the four treatment groups given C1I, there were 3 deaths, compared with the 11 rats not given C1I in which there were 7 deaths. The addition of C1I significantly (p = 0.04) reduced ICH mortality by inhibiting bleeding.

#### ICH semiquantitation (Part I)

**Figure 5:**
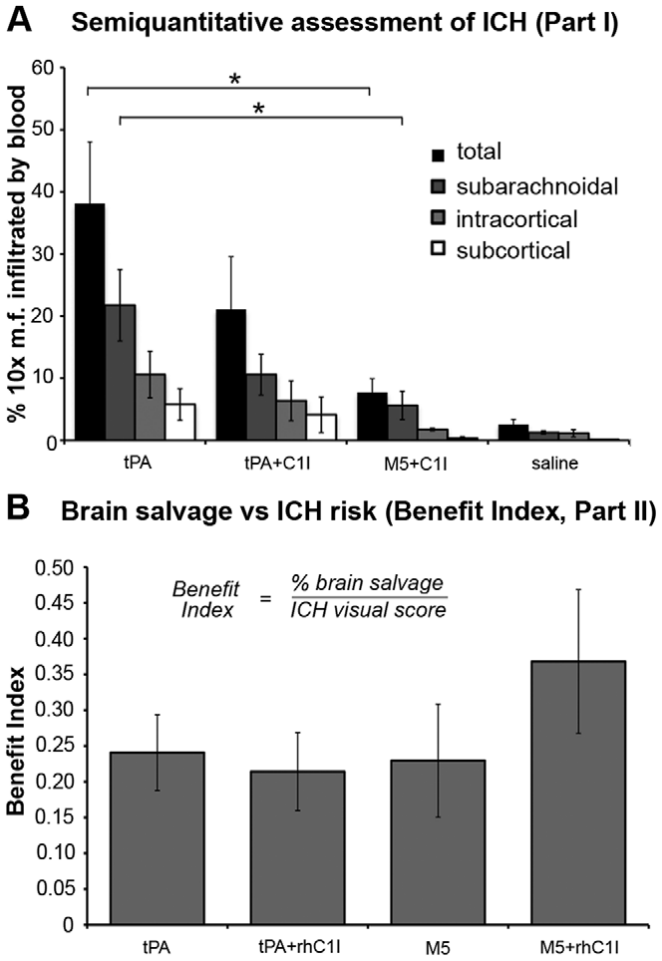
Semiquantitative ICH from Part I (A) and Benefit Index from Part II (B). A. ICH (%) by anatomical region in Groups 1, 2 (tPA) and 4 (M5+C1I). Group 3 could not be examined microscopically due to extensive hemorrhage. Extent of ICH in Group 4 was significantly (*p<0.05) less than in Group 1. B. Benefit Index defined as brain salvage divided by ICH complications. Brain salvage = control - post treatment ischemic volume/control ischemic volume. ICH complications = ICH score. The mean of the ratios and SEM for each treatment group is shown. Group 4 had the best Benefit Index but the difference from the other groups did not reach statistical significance (p = 0.29).

The inspection of microscopic fields at 10x magnification showed differences in the amounts of blood infiltration among Groups 1, 2 and 4 (Figure 5A, Table 1). As mentioned above, Group 3 (M5 alone) could not be included in this analysis due to the large extent of the bleeding, as anticipated by the absence of tcM5 inhibition in rat plasma (Figure 1). By contrast, with the addition of C1I (Group 4), only 7.7%±2.2 of the microscopical fields were found infiltrated by blood, which was was significantly (p = 0.02) less than with tPA (Group 1) in which 38.1%±9.9 were infiltrated. With the addition of C1I (Group 2) bleeding was reduced to 21.1%±8.4 but this was insignificantly different from the others. Anatomically, surface infiltration predominated in all groups, and was least in Group 4, in which only 5.6%±2.3 surface fields were infiltrated, compared to 21.7%±5.7 in Group 1 (p = 0.015) and 10.6%±3.3 in Group 2 (p = 0.38). Similarly, at the cortex and striatum Group 4 showed the least infiltration compared to Group 1 and 2, although these differences were not statistically significant.

#### Part II (ICH visual score)

ICH was not seen in any of the control rats (Groups 5 and 6), indicating that it was related entirely to the effect of thrombolysis in this model, and none of the control rats died. The ICH visual score was most severe with tPA (2.5±0.6) and M5 alone (2.3±0.5), in which one rat died associated with profuse bleeding from the transected tail wound, indicating a systemic hemorrhagic state (Table 1). With adjunctive rhC1I (Group 4), ICH was again reduced (1.7±0.3). In Group 2, rhC1I also inhibited ICH (1.8±0.5), but this was associated with inhibition of fibrinolysis, as reflected by a ∼40% increase in ischemic volume.

#### Benefit Index

The Benefit Index defined as the ratio of brain salvage and bleeding complications (ICH) was greatest for Group 4, though the difference between it and Group 1 did not reach statistical significance (p = 0.29). The mean values and SEM are shown (Figure 5B).

## Discussion

TPA has remained the only thrombolytic available for clinical use in ischemic stroke since its approval in 1997 [2]. It is the standard against which new thrombolytics must be compared. In view of the well documented limitations of tPA, a new thrombolytic should optimally have a greater reperfusion rate than tPA, not have a similar dose limitation by ICH complications [24], [25]; have larger treatment window; and no blood brain barrier (BBB) disruption [26], [27] or neurotoxicity [8], [11].

In Part I of the present study, irreversible ischemia was used to evaluate differences in hemorrhagic complications of the thrombolytic regimens, especially the salutary effects of C1I. In order to enhance the hemorrhagic side effects, thrombolysis was delayed four hours after the ischemia. As a result, fatal ICH occurred in as many as 57% and 75% of rats with tPA and M5 respectively. Since the rat is relatively resistant to tPA, 10 mg/kg, more than ten-times higher than the human dose, is needed for efficacy [28], and is the dose used in most studies in the literature [26], [29]–[32]. The rat is even more resistant to human uPA, so 15 mg/Kg M5 was used [14].

C1I administration reduced ICH mortality with M5 to 17% of rats (Group 4), and significantly reduced blood infiltration in the microscopic fields (Figure 5A). This finding demonstrated the salutary effect of C1I on bleeding complications by M5 for the first time, which heretofore, had only been show *in vitro* as an effect on non-specific plasminogen activation by M5 during clot lysis [22], [23]. Importantly, the C1I inhibition of bleeding was not accompanied by any reduction in thrombolytic efficacy by M5, as shown in Part II by the equivalent ischemic volumes in Groups 3 and 4. This finding was also consistent with that obtained from clot lysis studies with M5 and C1I [22].

C1I also reduced fatal ICH to 25% with tPA (Group 2) and reduced blood infiltration in the microscopic fields, but not to a significant degree. In contrast to M5, this C1I effect was accompanied by a major inhibition of fibrinolysis, as evidenced by a ∼40% increase in ischemic volume by C1I compared to tPA alone, as shown in Part II. Therefore, the C1I inhibition of bleeding complications without impairing thrombolysis only occurred with M5, reflecting fundamental differences in these two plasminogen activators.

**Figure 6:**
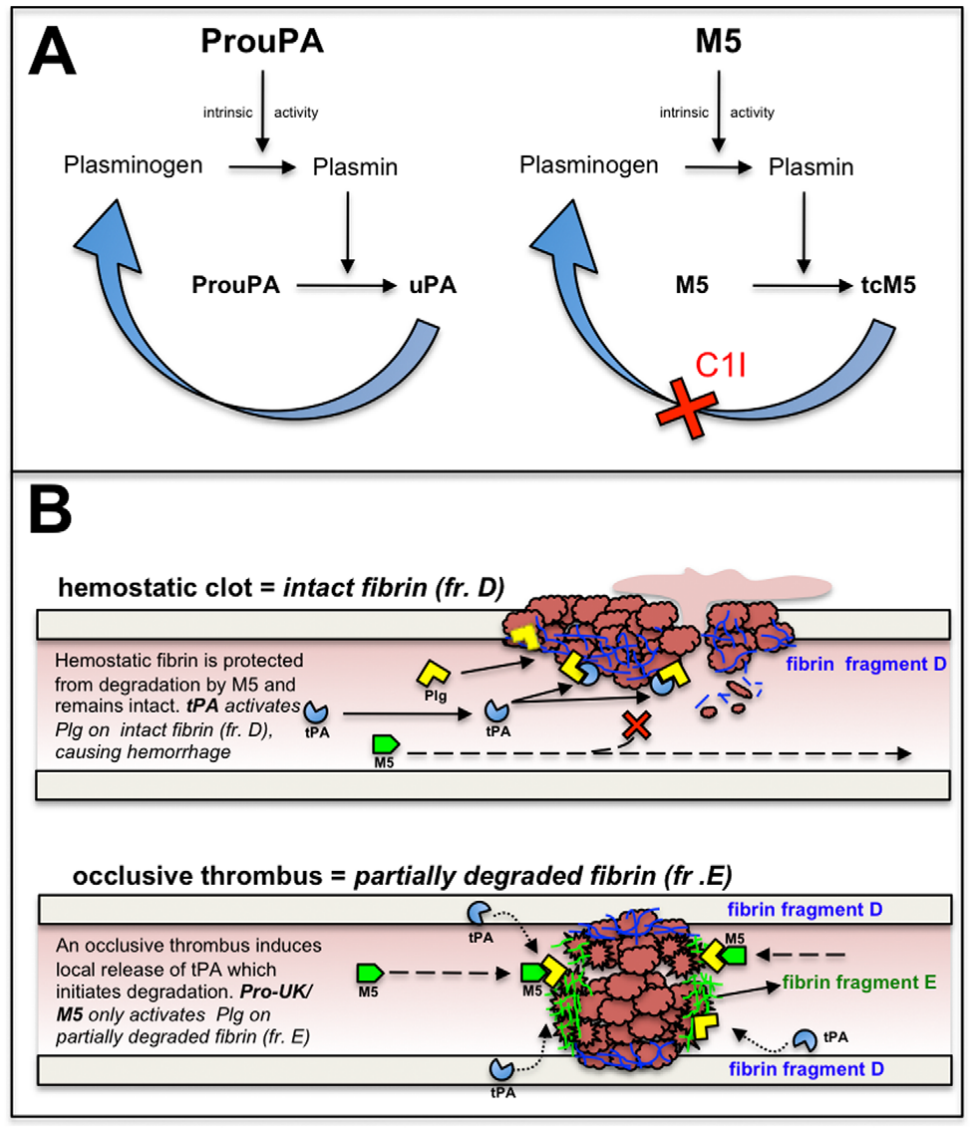
Illustration of ProuPA vs M5 at therapeutic concentrations in plasma and of the lysis of hemostatic vs occlusive fibrin by tPA and M5. A. At therapeutic concentrations of prouPA or M5, their intrinsic activities can activate plasminogen in plasma and the plasmin generated will then activate these proenzymes to their respective enzymes uPA and tcM5. The latter is irreversibly inactivated by C1I, which prevents the positive feedback, thereby preventing non-specific plasmin generation responsible for hemorrhagic side effects. B. Hemostatic fibrin, being protected from degradation physiologically, contains only the plasminogen binding site on the fibrin fragment-D domain of intact fibrin. At therapeutic concentrations, tPA, being free of its inhibitor PAI-1, will bind to an adjacent site to plasminogen resulting in its activation and bleeding from the degraded hemostatic site. Occlusive thrombus triggers the release of tPA from the vessel wall which initiates its degradation physiologically. This exposes new plasminogen binding site, particularly the high affinity site on fibrin fragment E domain with its three C-terminal lysine. Plasminogen binding to this site undergoes a conformational shape change which permits its activation by M5 causing lysis. TPA will also lyse the occlusive clot since other plasminogen binding sites remain. The two plasminogen activators are complementary and synergistic in their fibrinolytic mechanisms.

M5 is a proenzyme which requires plasma inhibitors, principally C1I, to prevent its non-specific activation to tcM5 (see Figure 6A). When this occurs, its fibrin-specificity is lost resulting in bleeding complication due to plasminemia. This C1I effect in preventing this non-specific plasminogen activator effect is analogous to that of plasminogen activator inhibitor-1 (PAI-1) in controlling the physiological function of prouPA [33]. However, the endogenous concentration of C1I is much greater than that of PAI-1 [15], and can be further supplemented by exogenous C1I, as shown in the present study. In addition, C1I is a weaker inhibitor than PAI-1 [34], and, therefore is unable to not interfere with the more efficient fibrin-dependent plasminogen activation [23], explaining why thrombolysis was not inhibited by C1I. By contrast, tPA is an enzyme in both its single and two-chain forms so that its fibrin-specific properties are not dependent on plasma inhibitors. Furthermore, tPA and prouPA are complementary and synergistic [35] in their fibrin-dependent plasminogen activation, which also helps explain a difference in their effect on hemostatic fibrin. TPA binds to fibrin at a site on the D-domain of intact fibrin where it activates plasminogen bound to this site [36], [37]. This so-called ternary complex promotes plasminogen activation by tPA as much as 1000-fold [38]. ProuPA/M5 has no fibrin affinity but has a high substrate affinity [37], which is to plasminogen bound to the fibrin E-domain [39], which is found only on degraded fibrin. Hemostatic fibrin, being protected from degradation, contains the D but not the E-domain (see Figure 6B). Therefore, unlike tPA, M5 spares hemostatic fibrin, as previously reported [20], but this is highly dependent on the prevention of non-specific conversion to tcM5. Therefore, M5 is highly dependent on plasma inhibitors (C1I), which is not the case for tPA. Bleeding by tPA is believed to be principally related to its lysis of hemostatic fibrin due to its fibrin binding site being on intact fibrin like hemostatic fibrin [40] (Figure 6B).

The tPA dose in Part II was reduced to 5 mg/K due to the high ICH rate in Part I. In addition, rhC1I (equivalent in per unit activity to plasma C1I) was substituted for C1I purified from human plasma. As in Part I, M5 alone (Group 3) induced severe ICH and profuse re-bleeding from the transected tail, reflecting a systemic hemorrhagic state, results consistent with the absence of tcM5 inhibitory activity in rats (Figure 1). Pre-treatment with rhC1I virtually eliminated M5 bleeding, and this group (Group 4) was the only one with significant improvement in neurofunction after thrombolysis (Figure 3A). The inhibition of ICH by C1I was semiquantitated by a standardized visual score, which showed significant (p = 0.02) reduction in overall ICH in Group 4 compared with Group 1 or 3 (Figure 5A).

The post-treatment ischemic volumes were comparable in Groups 1, 3, and 4, showing that their fibrinolytic effects were the same, whereas the addition of rhC1I to tPA(Group 2) resulted in a ∼40% increase in ischemic volume reflecting inhibition of tPA mediated fibrinolysis by rhC1I, in contrast to its effect on M5. The edema volume associated with tPA was consistent with its known disruption of the BBB, a side effect not shared by uPA [41]. Interestingly, the addition of rhC1I reduced the edema volume by almost 40% (Figure 3B, Table). Since no direct effect of rhC1I on ischemia-mediated BBB disruption was seen in placebo Group 6 vs 5, the rhC1I effect was related to tPA-induced edema formation, suggesting that complement activation may have been involved. It has been reported that tPA activates the complement pathway *in vivo*, generating anaphylatoxins in patients treated with tPA for coronary thrombosis [42].

Since the net clinical benefit of thrombolysis corresponds to the positive effects of reperfusion and the negative effects of ICH, an attempt was made to calculate a “Benefit Index” by combining the two (Figure 5B). In an analogous way the Clinical Utility Index has been recommended for drug development [43]. The Benefit Index was greatest for Group 4, though this did not reach statistical significance (Figure 5B), and correlated with the neuroscore improvement in Group 4 (Figure 3A).

In conclusion, a prouPA mutant, M5, was tested in two rat stroke models against the effects of tPA, the current standard. The ICH and ICH mortality were greatest with M5 and tPA, but were significantly (p = 0.04) reduced by the addition of C1I. No ICH was seen in either of the placebo groups showing that in this model, ICH was a side effect of thrombolysis. In Part II, the ischemic volume reductions by tPA, M5, and M5+rhC1I were equivalent, allowing their complication rates to be compared. ICH was again greatest with M5 and tPA, and the addition of rhC1I to M5 prevented this without impairing thrombolysis by M5. By contrast, when rhC1I was combined with tPA, both ICH and thrombolysis were inhibited, reflecting an important difference in the mechanisms of action of these two plasminogen activators.

The findings provide the first demonstration that exogenous C1I prevents bleeding complications by M5 without interfering with thrombolysis, an effect which heretofore had been shown only *in vitro* as an inhibition of non-specific plasminogen activation by M5 during clot lysis [22], [23]. Since thrombolytic efficacy is currently limited by dose dependent ICH (requiring tPA dose to be restricted to 0.9 mg/Kg), a thrombolytic with a selective antidote for ICH would not have this limitation and could improve thrombolytic efficacy in stroke to an unprecedented degree.

## Materials and Methods

### Ethical statement

Male adult Sprague-Dawley rats (Harlan, Italy) weighing 270–350 g were used in this study. All animal experimental procedures were approved and carried out in accordance to European Community Council Directive 86/609/EEC (November 24, 1986), Italian Ministry of Health and University of Turin institutional guidelines on animal welfare (law 116/92 on Care and Protection of living animals undergoing experimental or other scientific procedures; authorization number 17/2010-B, June 30, 2010) and *ad hoc* Ethical Committee of the University of Turin. Free access to food and water was maintained and all efforts were made to minimize suffering and limit the number of animals used.

### Experimental plan

To help evaluate the species suitability of the rat for this study of M5, rat plasma was compared with other species before performing any surgical procedure. TcM5 (10 µg/mL) was incubated (37°C) 1h in plasma from rat, human, dog, guinea pig and rabbit. The quenching of tcM5 activity was monitored over 1 h with uPA amidolytic substrate (S2244). After 1 h incubation, the plasmas were examined by zymography on plasminogen enriched casein plates as previously described [20], a method sensitive to plasminogen activator:inhibitor complexes. Male adult Sprague-Dawley rats (Harlan, Italy) weighing 250–350 g were used to compare therapeutic fibrinolysis by tPA or M5+/− C1I following middle cerebral artery (MCA) occlusion. In Part I (n = 35), we evaluated intracranial hemorrhage (ICH) complications with a model of irreversible ischemia by permanently occluding the MCA. Survival and anatomical findings at 24 hours in surviving animals were monitored. In Part II (n = 31), thromboembolic MCA occlusion was used and ischemic volume, edema volume, ICH, and functional recovery were evaluated at 24 hours. Rectal temperature was maintained at 36.5–37.5°C throughout the experiment with a heating pad (Ugo Basile, Varese, Italy).

### Materials

M5 was expressed in *Escherichia coli* as previously described [20] and obtained from Primm (Milan, Italy). C1I purified from human plasma (Berinert P®) was kindly supplied by Behring GmbH (Marburg, Germany); rhC1I was kindly supplied by Pharming (Leiden, The Netherlands), and tPA (Actilyse®, Boehringer Ingelheim, Germany) was a generous gift of dr. Bergui (Department of Neuroscience, University of Turin).

#### Treatment protocols

Infusions of tPA or M5 were administered by pump (KD Scientific, Holliston, MA, USA) through the right femoral vein under 2% isoflurane anesthesia with 10% of the total dose given as a bolus at outset and the remainder over 30 minutes. The tPA dose for part I was 10 mg/Kg, the dose used in most published studies in rat stroke [26], [29]–[32] and was given 4 h after the MCA occlusion. Because of the high incidence of fatal bleeding complications in Part I, the tPA dose was reduced to 5 mg/Kg for Part II and all treatments were administered 2 h after occlusion. The M5 dose of 15 mg/Kg was used in both Part I and II, and was estimated from results of comparative clot lysis data in rat plasma; in a preliminary experiment, this dose was found effective to dissolve clot and re-establish adequate blood flow as confirmed by laser doppler flowmetry profile (LDF, see Figure S1 A online).

The C1I (100 IU/kg) in part I or rhC1I (180 IU/kg) in part II was administered as a bolus before fibrinolysis. The per IU activities of C1I and rhC1I were shown to be equal (unpublished observations).

### Experimental groups

In both Part I and II, before MCA occlusion, the animals were randomly assigned to the following 6 groups through computer-generated randomization schedules: (1) tPA; (2) tPA+C1I; (3) M5; (4) M5+C1I; (5) saline control; (6) saline+C1I. Group 3 was allotted the fewest rats since the absence of endogenous C1I activity in the rat (Figure 1) made hemorrhagic complications so likely that little variability was anticipated.

#### Part I. Permanent ischemia to evaluate hemorrhagic complications (35 rats)

The MCA was cauterized by the method described by Renolleau [44]. Rats were anesthetized with isoflurane (4% during induction, then maintained with 1.5%), in a mixture of 30∶70 O_2_/N_2_O delivered with a face mask throughout the surgery duration. Briefly, under an operating microscope (Carl Zeiss Inc., Jena, Germany), a midline incision of the head was performed, the temporal muscle dissected and the temporal bone exposed; a burr hole was drilled very close to the zygomatic arch and the left MCA was identified. The MCA main branch was then electrocoagulated close to its origin at the junction with the olfactory branch. Thereafter, a median incision was made in the neck to expose the left common carotid artery (CCA), which was transiently occluded by a clip in order to reduce infarction size variability due to anastomoses in the MCA territory. After 90 minutes, the clip was removed. Cortical blood flow variations from ischemia were not measured in this part, as MCA was cauterized under visual control and successful occlusion was confirmed by progressive whitening of the cortex. Infusions were initiated 4h later.

### Neuropathological examination

After 24 h, the surviving rats were given a lethal dose of chloral hydrate and then perfused through the left ventricle with saline followed by fixative (paraformaldehyde 4% in 0.1 M phosphate buffer, PB, pH 7.4). Brains were then removed, post-fixed for 3 h in the same paraformaldehyde solution, infiltrated overnight in 30% sucrose in PB for cryoprotection and stored at −20°C. Brains were sectioned on the cryostat in 50 µm-thick serial sections; every sixth slice was mounted on gel-coated slides and stained with cresyl violet for histological evaluation and semiquantitation of hemorrhagic infiltration.

### Semiquantitative analysis of bleeding

In order to measure the extent of hemorrhagic infiltration of the brain, in the Groups 1, 2 and 4 (this was not possible for Group 3 due to massive hemorrhagic destruction of tissue) every sixth section was inspected under the microscope (at 10x magnification) and compared to saline group (Group 5; Group 6 was not included in analysis as no evidence of macroscopical hemorrhage was found, similarly to Group 5). For each section, the total number of microscopic fields (m.f.) was inspected (mean 754 fields ±15 per animal) and the number infiltrated by blood was counted, with reference to location (cortical surface, cerebral cortex or striatum). The data were expressed as percentage of fields infiltrated by blood in each area.

#### Part II: Thromboembolic ischemia (31 rats)

All infusions (tPA 5 mg/kg or M5 15 mg/kg or placebo) were administered 2 h post occlusion. RhC1I (180 IU/kg) was administered as a bolus before the infusions. Ischemia was induced by injection of autologous blood clots in suspension into the internal carotid artery (ICA), as described by Busch [45]. In order to prepare blood clots for embolism, femoral arterial blood from a donor rat was collected into a 20 cm long PE-50 catheter and retained for 2 hours at room temperature and subsequently at 4°C for 22 hours to allow clot formation to go to completion. Clots were pushed out of the catheter with a saline-filled syringe, rinsed several times in a Petri dish containing phosphate-buffered solution (PBS, pH 7.4), in order to remove blood cells and obtain a white clot, then inspected under the microscope to select fibrin-rich fragments. These fragments were cut into 2 mm-long pieces and transferred into a solution containing 1 mg/mL albumin in PBS to allow clot retraction. Approximately 2 hours later, 20 fibrin-rich fragments were drawn up in the albumin solution in one meter-long PE50 catheter, taking care to maintain ∼3 cm distance between clots in order to keep them apart, and all embolized into MCA origin.

### Surgical procedures

Rats were anesthetized with isoflurane (4% during induction, then maintained with 1.75%), in a mixture of 30∶70 O_2_/N_2_O delivered with a face mask throughout the surgery duration. After a longitudinal incision of 2 cm in length in the midline of the ventral cervical skin, left CCA, internal (ICA) and external carotid artery (ECA) were carefully dissected and exposed, avoiding any damage to the adjacent vagus nerve. Inferior thyroid and occipital arteries, branching from ECA, were visualized and cauterized; the distal portion of ECA was ligated and cut along with the terminal lingual and maxillary artery branches, and the carotid bifurcation identified. ICA was dissected cranially up to the origin of pterygopalatin branch, which was ligated using a 6/0 suture. A 5-0 silk suture was loosely tied around the origin of ECA, and then the CCA and ICA were temporarily clamped using microvascular clips. A PE-50 catheter containing blood clot suspension with a modified 0.3 mm outer diameter was introduced into the ECA stump through a small puncture and advanced 1–2 mm beyond carotid bifurcation; the 5/0 suture was tightened around the catheter to prevent backflow bleeding. The clip around ICA was removed and clots injected within 30 s, while CCA was still occluded. At the end of injection, the catheter was withdrawn, the ECA stump was ligated and the CCA clip removed, so that blood pressure could push clots cranially. The wound was then closed; the rat was allowed to completely recover from anaesthesia and returned to its cage. The surgery was complete in approximately 30 minutes.

### Laser Doppler flowmetry (LDF)

Occlusion was confirmed by LDF using Biopac LDF100C (Biopac Systems, CA, USA), using an optical fibre probe positioned 0.5 mm above the dural surface, 1 mm posterior and 5.5 mm lateral to the bregma. Recordings of cortical blood flow (CBF) in the MCA territory were started 30 minutes before occlusion and CBF changes were expressed as percentage of pre-ischemic value; a 70% drop from the baseline levels indicated successful occlusion. Time profile of recanalization was not further investigated as LDF was previously shown not to reliably predict thrombolysis-mediated reperfusion [46].

### Re-bleeding

The rat tail was transected 5 mm from the tip and allowed to stop bleeding before thrombolysis was initiated. Re-bleeding from this site, reflecting lysis of hemostatic fibrin, was monitored.

### Functional neuroscore

Functional outcome was tested at 2 and 24 h by observers blinded to the pharmacological regimen. A 5 point scale described by Longa [47] was used (grade 0: no neurological deficit; grade 1: failure to fully extend left forepaw; grade 2: contralateral circling; grade 3: contralateral falling; grade 4: absence of spontaneous movement or unconsciousness). The neuroscore was also used to further confirm that successful thrombotic occlusion had been achieved, since the existence of initial neurological deficit is a reliable predictor for successful occlusion of the MCA [48], [49]; only animals grade 2 or higher at 2 h were included in the study. Exclusion of animals took place before assignment into the various treatment groups.

### Determination of infarct and edema volumes

At 24 h, the animals were euthanized; brains were removed, cooled on ice, and then coronally cut into 2 mm-thick sections using a tissue slicer, starting from 2 mm caudal to the frontal tip. Sections were immediately stained with 2% 2,3,5-triphenyltetrazolium chloride (TTC, Sigma, St. Louis, MO) at 37°C for 10 minutes, then fixed in 4% phosphate-buffered formalin as previously described [50]. Each slice was examined for subarachnoid hemorrhage. Slices were scanned with a Coolpix camera (Nikon, Sesto Fiorentino, Italy); infarct volume and brain edema were measured using NIH ImageJ analysis software (available at http://rsb.info.nih.gov/ij/). Ischemic volumes were calculated as the sum of infarcted area (in mm^2^) multiplied by slice thickness (∼2 mm). Edema volumes were calculated by subtracting the contralateral hemisphere volume from the ischemic hemisphere volume; for edema correction, the equation ischemic volume * contralateral hemisphere/ipsilateral hemisphere volume was used, as previously described [51]. Seven 2-mm thick slices were measured for each brain.

### ICH visual scoring

The severity of ICH was assessed as in Choudhri et al. with some modifications [52]. Briefly, TTC-stained sections were inspected by a blinded observer and the degree of ICH was given a score based on maximal hemorrhage diameter measured on any of the sections using NIH ImageJ software (ICH score 1: no hemorrhage; score 2: <1 mm; score 3: 1 to 2 mm; score 4: 2 to 3 mm; score 5: >3 mm).

### Benefit index

The percentage of brain salvage (control ischemic volume - post treatment ischemic volume/control ischemic volume) and the ICH score were used to calculate a Benefit Index, as an overall measure of treatment success. Similarly, a “Clinical Utility Index” has been advocated as useful for drug development decisions [43].

### Statistical analysis

Data are presented as mean±standard error of the mean (SEM); all statistical analysis was conducted using SPSS package (version 18, SPSS Inc., Chicago, IL, USA); differences were considered statistically significant when p<0.05. The Shapiro-Wilk and the Levene median tests were initially used to describe the data distribution and to determine the equality of the variances, respectively. In Part I, mortality rates in the groups were compared using the Fisher's exact probability test (two-tailed). Non parametric approach was adopted to compare differences in bleeding (Kruskal Wallis and Mann Whitney statistics, when appropriate). In part II, the one-way ANOVA was applied to determine for overall significant differences in ischemic and edema volumes among groups, and to compare Benefit Indices. Post-hoc analysis were conducted when p<0.05 by Fisher's protected least significant difference (PLSD) test. Non-parametric paired t-test (Wilcoxon signed rank test) was adopted to evaluate differences in motor behavior in each group between 2 h and 24 h.

## Supporting Information

Figure S1 **LDF variations after M5 15 mg/kg infusion.** A**.** Following clot injection, MCA occlusion is confirmed by ∼75% drop of LDF signal. Compared to vehicle group, M5 15 mg/kg infusion adequately re-establish blood flow in MCA, as confirmed by progressive rise of LDF over the next 60 minutes. Recanalization was effective starting 30 to 40 minutes after MCA occlusion was achieved (*p<0.05, **p<0.01). B: hemorrhagic brains from group 3 (M5 alone), part I. Intense bleeding in this group reflected aspecific activation of M5 in absence of C1-inhibitor and caused 75% mortality due to massive hemorrhage, starting 30 minutes to 4 hours after treatment.
(TIF)
