# Supplementary figures and images for: Mutant Prourokinase with Adjunctive C1-Inhibitor Is an Effective and Safer Alternative to tPA in Rat Stroke
Source: PLoS One. 2011 Jul 14;6(7):e21999. doi: 10.1371/journal.pone.0021999 (PMC3136496; doi:10.1371/journal.pone.0021999)

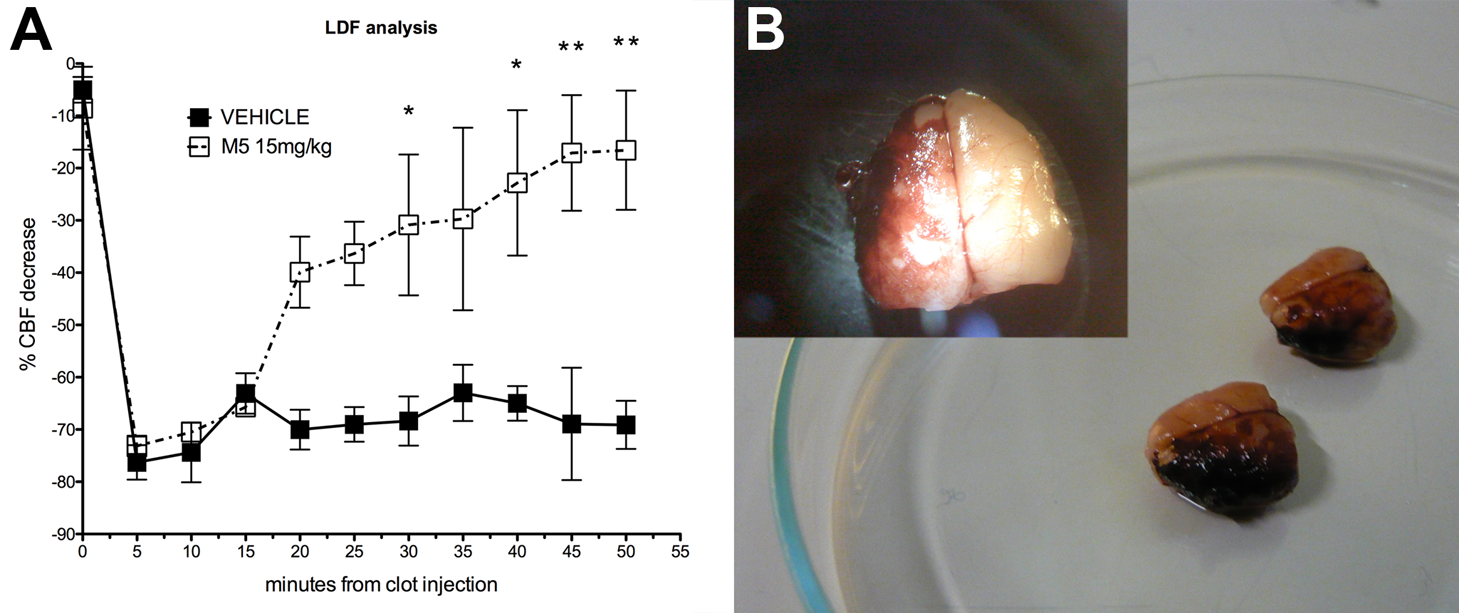

Supplement: Figure S1 — LDF variations after M5 15 mg/kg infusion. A. Following clot injection, MCA occlusion is confirmed by ∼75% drop of LDF signal. Compared to vehicle group, M5 15 mg/kg infusion adequately re-establish blood flow in MCA, as confirmed by progressive rise of LDF over the next 60 minutes. Recanalization was effective starting 30 to 40 minutes after MCA occlusion was achieved (*p<0.05, **p<0.01). B: hemorrhagic brains from group 3 (M5 alone), part I. Intense bleeding in this group reflected aspecific activation of M5 in absence of C1-inhibitor and caused 75% mortality due to massive hemorrhage, starting 30 minutes to 4 hours after treatment. (TIF) [file pone.0021999.s001.tif]
